# Probing Biointerfaces with QCM‑D and Electrochemistry: Opportunities and Challenges toward Integrated EQCM‑D Biosensing

**DOI:** 10.1021/acsmeasuresciau.6c00010

**Published:** 2026-02-23

**Authors:** Shu-Hong Lin, Yijie Hsu, Shyh-Chyang Luo

**Affiliations:** Department of Materials Science and Engineering, National Taiwan University, No. 1, Sec. 4, Roosevelt Road, Taipei 10617, Taiwan

**Keywords:** electrochemical quartz crystal microbalance with dissipation monitoring (EQCM-D), multimodal biosensing, biointerface, cellular behavior, viscoelastic characterization, hydration and ion effects, gravimetric−electrochemical coupling, spatiotemporal analysis

## Abstract

Biosensing has garnered significant attention due to the demand for early disease diagnosis and the interest in biological interfaces where gravimetric response, hydration, mechanical deformation, and charge transfer are intrinsically coupled and dynamically evolving. Quartz crystal microbalance with dissipation monitoring (QCM-D) and electrochemical techniques provide complementary access to interfacial mechanical and electrical properties, yet each alone faces limitations in interpreting the complexity. This review summarizes recent advances in QCM-D- and electrochemistry-based biosensing, discussing strategies for their coupling, with a particular focus on electrochemical quartz crystal microbalance with dissipation monitoring (EQCM-D). By enabling simultaneous gravimetric, viscoelastic, and electrochemical measurements at the same interface, EQCM-D offers unique opportunities for real-time correlation of biointerfacial processes. We further highlight emerging challenges in data interpretation arising from the active roles of water and ions and discuss future perspectives toward correlative multimodal EQCM-D platforms for quantitative biosensing and bioelectrochemical studies.

## Graphical abstract

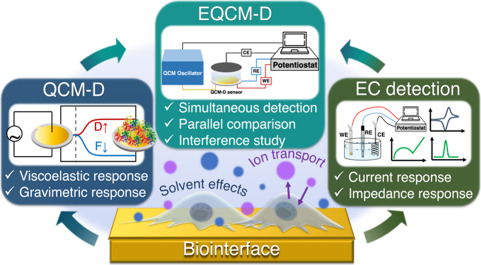

## 1 Introduction

In recent years, biosensing has become a rapidly growing field driven by the booming demand for early disease diagnosis, point-of-care testing, and the analysis of complex clinical samples.^1,2^ In addition to the quantitative analysis of the targets, biosensing has emerged as an essential approach for probing biological systems, enabling the investigation of biomolecular interactions, cellular behaviors, and biointerfaces in real time.^3,4^ Accordingly, modern biosensing platforms are increasingly expected to provide interfacial information that links biological responses to underlying physical and chemical processes.

A wide range of biosensing techniques has been reported, including surface-enhanced Raman scattering (SERS), surface plasmon resonance (SPR), quartz crystal microbalance with dissipation monitoring (QCM-D), as well as electrochemical and fluorescence-based methods.^5–8^ However, achieving reliable biosensing remains challenging due to the intrinsic complexity of biological systems. Biomolecular interfaces are typically soft, highly hydrated, and dynamically evolving, where adsorption and desorption, structural rearrangements, hydration, and charge transfer often occur simultaneously.^9,10^ These coupled and time-dependent processes make it difficult to interpret biosensing signals using a single measurement modality.

Among these techniques, QCM-D and electrochemical methods are particularly attractive due to their ability to assess interfacial behaviors. QCM-D provides intuitive and sensitive gravimetric and viscoelastic information, enabling real-time monitoring of gravimetric response and mechanical properties at interfaces.^11,12^ Electrochemical methods, on the other hand, offer quantitative access to interfacial charge transfer, capacitance, and reaction kinetics.^13,14^ Owing to their complementary strengths, QCM-D and electrochemical techniques have been combined through sequential or parallel coupling, as well as through direct integration into a single platform, known as electrochemical quartz crystal microbalance with dissipation (EQCM-D). By enabling simultaneous gravimetric, viscoelastic, and electrochemical measurements at the same interface, EQCM-D provides a unique opportunity to directly correlate mechanical and electrochemical responses in real time.

In this review, we summarize recent advances in QCM-D and electrochemical techniques, with a particular focus on their integration into EQCM-D platforms. While prior reviews have cataloged the technical configurations of these systems, this work distinguishes itself by first establishing the complementary nature of these sensing modalities (Section 2) and specifically addressing the active roles of water and ions, which are often overlooked as background, in modulating coupled readouts (Section 5). In addition to surveying representative examples in protein, DNA, and vesicle sensing (Sections 3 and 4), we emphasize the challenges in signal interpretation and provide a methodological framework (Section 6) for deconvolving genuine biorecognition from electrochemical artifacts through spatiotemporal analysis. Finally, we discuss emerging trends toward correlative multimodal platforms for studying complex bioelectrochemical interfaces (Section 7).

## 2 QCM-D for Biosensing

### 2.1 Fundamentals of QCM-D

Quartz crystal microbalance (QCM) operates based on the converse piezoelectric effect, in which an alternating electric field induces thickness-shear oscillations of a quartz crystal.^15,16^ Perturbations at the sensor surface modify the resonance behavior of the crystal, leading to shifts in its resonance frequency.^17,18^ Under ideal conditions, these frequency shifts are linearly related to mass loading, as described by the Sauerbrey equation.^19^ This formulation assumes a thin, rigid, and homogeneously coupled adlayer in air, allowing frequency changes to be interpreted as dry mass adsorption.

However, the Sauerbrey model is rarely satisfied in liquid-phase biosensing.^20^ Soft and hydrated biological adsorbents, such as proteins, cells, and extracellular vesicles (EVs), undergo viscoelastic deformation and interact strongly with the surrounding solvent during oscillation, resulting in energy dissipation that cannot be interpreted by frequency shifts alone.^21,22^ To account for this nonideal interfacial behavior, dissipation monitoring was incorporated into QCM measurements, resulting in QCM with dissipation (QCM-D).^23^ Unlike the original QCM, which was primarily limited to gas-phase sensing, QCM-D breaks through this constraint by enabling precise measurements in the liquid phase, making it an essential tool for probing dynamic biointerfacial processes. By simultaneously monitoring the real-time shifts in resonance frequency (Δ*F*) and energy dissipation (Δ*D*), QCM-D senses coupled mass, hydration, and mechanical responses at biointerfaces, therefore enabling the investigation of dynamic interfacial processes, such as molecular binding, cell adhesion, and vesicle deformation.

### 2.2 Typical Applications of QCM-D

QCM-D offers a distinctive advantage in biosensing by providing real-time, in situ access to interfacial processes during biomolecular, vesicular, or cellular attachment. Simultaneous monitoring of frequency (Δ*F*) and dissipation (Δ*D*) enables label-free detection while also capturing viscoelastic information, making QCM-D particularly suitable for soft, hydrated biological systems.^24,25^ These capabilities have enabled not only quantitative sensing but also mechanistic interrogation of interfacial binding, adhesion, and deformation processes.

#### 2.2.1 Ligand–Receptor Orientation Tests

The major applications of QCM-D are summarized in Figure 1. One representative example is ligand–receptor conjugation, where target orientation and surface organization critically determine binding efficiency. Behan et al. systematically investigated protein-modified nanoparticles with controlled terminal orientation, grafting density, and receptor density.^26^ QCM-D measurements revealed that C-terminally oriented proteins produced larger frequency shifts than nonspecific corona-covered ones and PEG-modified nanoparticles, which exhibited antifouling property, consistent with enhanced binding accessibility (Figure 1a). This study illustrates how QCM-D can visualize binding behaviors and discriminate between surface-limited and transport- or affinity-limited regimes.

**Figure 1:**
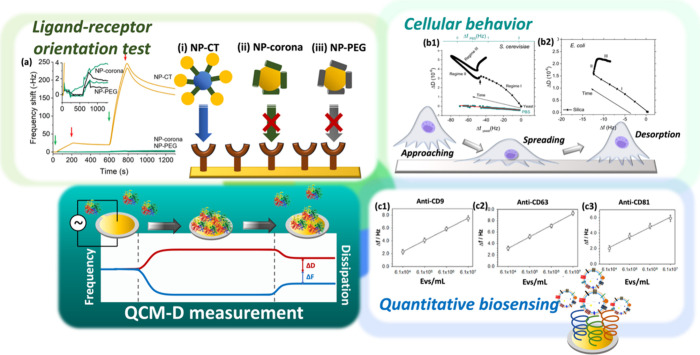
Working principle and major applications of QCM-D, including (a) ligand–receptor orientation test, (b) cellular behavior, and (c) quantitative biosensing. (a) Binding profiles obtained for NPs with (i) grafted (NP-CT), (ii) adsorbed ApoE (NP-corona), and (iii) grafted PEG on immobilized ApoE R2 substrate. Reproduced with permission from ref 26 Copyright 2023, the Authors. Published by American Chemical Society. This is an open-access article under the terms of the Creative Commons Attribution License (CC BY 4.0). (b1) The df plot for *S. cerevisiae* (yeast) on silica for a period of 48 h; (b2) the df plot for *E. coli* adhesion on silica for a period of 24 h. Reproduced with permission from ref 27 Copyright 2020, American Chemical Society. (c) The calibration lines of anti-CD9 (c1), anti-CD63 (c2), and anti-CD81 (c3) antibodies against different concentrations of lung cancer cell-derived Evs Reproduced with permission from ref 28 Copyright 2023, the Authors. Published by American Chemical Society. This is an open-access article under the terms of the Creative Commons Attribution License (CC BY 4.0).

#### 2.2.2 Cellular Behaviors

Beyond molecular recognition, QCM-D has been widely applied to study cell adhesion, which plays a crucial role in cellular communication, growth, and cell–material interactions.^29,30^ Conventional methods such as centrifugation,^31^ atomic force microscopy, and fluorescence-based techniques^32^ often rely on external forces or labels and typically probe only end point behavior. In contrast, QCM-D enables continuous, noninvasive monitoring of cell attachment and detachment dynamics through coupled Δ*F*–ΔD responses.

Yongabi et al. employed QCM-D to compare the adhesion behavior of eukaryotic ( *S. cerevisiae* ) and prokaryotic ( *E. coli* ) cells on different surfaces.^27^ Distinct dissipation–frequency (D–F) trajectories were observed, reflecting differences in cell stiffness, surface energy, and interfacial contact. Importantly, analysis of D–F plots allowed the adhesion process to be segmented into multiple regimes. For yeast on silica (Figure 1b1), early adhesion was accompanied by decreasing D–F slopes (Regime I), indicative of cytoskeletal stiffening, followed by a linear regime corresponding to cell spreading (Regime II). Subsequent detachment exhibited coupled increases in frequency and decreases in dissipation, while a final rise in dissipation at nearly constant frequency suggested viscoelastic restructuring of the residual surface-bound material (Regime III).

In contrast to the flat adhesion of yeast, *E. coli* exhibits a different behavior on silica (Figure 1b2). The unified slope in Regime I means adhesion without formation. In Regime II, the slightly increasing frequency implies the partial detachment. However, the constantly increasing dissipation indicates the loose and viscoelastic coupling with the substrate, where the flagella, fimbriae, and pili on the cell membranes form a hydrated film at the interface. Such analyses highlight that QCM-D responses cannot be interpreted solely in terms of gravimetric response but instead encode dynamic mechanical remodeling at the interface.

#### 2.2.3 Quantitative Biosensing

In addition to mechanistic studies, QCM-D has been adopted for quantitative biosensing. For example, Wang et al. developed a CNT–PEG-coated phenylboronic acid hydrogel sensor for glucose detection in saliva, achieving high sensitivity and selectivity through multivalent glucose binding and effective antifouling.^33^ Pohanka immobilized Fab fragments of antifibrinogen antibodies on gold-coated QCM sensors, achieving a detection limit of 0.075 g/L with strong correlation to ELISA measurements.^34^

QCM-D has also garnered significant interest in the detection of extracellular vesicles (EVs), motivated by their role in early cancer diagnostics. EV membranes present multiple surface antigens, enabling signal amplification through antibody–antigen interactions. EVs constitute a particularly demanding system for QCM-D analysis due to their soft, highly hydrated, and deformable nature. Kowalczyk et al. compared anti-CD9, anti-CD63, and anti-CD81-functionalized QCM-D and SPR sensors for EV detection, achieving limits of detection down to 0.6–1.8 × 10^4^ particles/mL (Figure 1c).^28^ Beyond sensitivity, QCM-D revealed antibody-dependent differences in EV binding affinity. Moreover, the decreasing slope of Δ*D* versus Δ*F* with increasing EV concentration suggested progressive vesicle packing and deformation, leading to a mechanically softer interfacial layer. This example highlights the unique strength of QCM-D in correlating analytical signals with nanoscale mechanical behavior.

These applications demonstrate that QCM-D signals reflect not only simple adsorption but also coupled mass, hydration, and mechanical effects, highlighting the analytical potential of QCM-D. However, the complexity implies the need for cautious interpretation in complex biological systems, particularly when considering viscoelastic contributions for accurate quantification.

### 2.3 Limitations of QCM-D

Despite its versatility in label-free biosensing, QCM-D faces inherent limitations when applied to complex biological systems. Owing to its universal sensitivity to mass coupled to the oscillating surface, QCM-D responses in liquid environments inevitably include contributions from solvent-associated effects, such as swelling and hydration layers.^35^ Furthermore, the rolling, sliding, deformation, and nonhomogeneous adsorption of adsorbents during the oscillation can cause additional responses.^27,36^ These contributions are intrinsic to the measurement principle and cannot be readily excluded, particularly for soft and hydrated biological assemblies, thereby complicating signal interpretation, chemical specificity, and selectivity.

Additionally, practical considerations further limit the applicability of QCM-D in specific sensing scenarios. Typical measurements require relatively large sample volumes and extended equilibration times to achieve stable baselines, which may hinder rapid analysis, high-throughput detection, or applications requiring significant matrix interference mitigation. These limitations motivate the integration of complementary techniques that provide orthogonal signal modalities and improved operational efficiency.

## 3 Electrochemical Biosensing

To address QCM-D limitations, electrochemical (EC) techniques offer selective, rapid, and quantitative readouts that are primarily insensitive to solvent-coupled mass effects. The common EC setup is a three-electrode system composed of a working electrode (WE), a reference electrode (RE), and a counter electrode (CE), as pictured in the scheme of Figure 2. Whereas QCM-D monitors changes in frequency and viscoelasticity at the sensor surface, EC methods detect electron-transfer events near electrodes, generating signals in the form of current, potential, or impedance.^37^ Among these, voltammetry and electrochemical impedance spectroscopy (EIS) are the most widely adopted in biosensing and exhibit the potential to couple with QCM-D.

**Figure 2:**
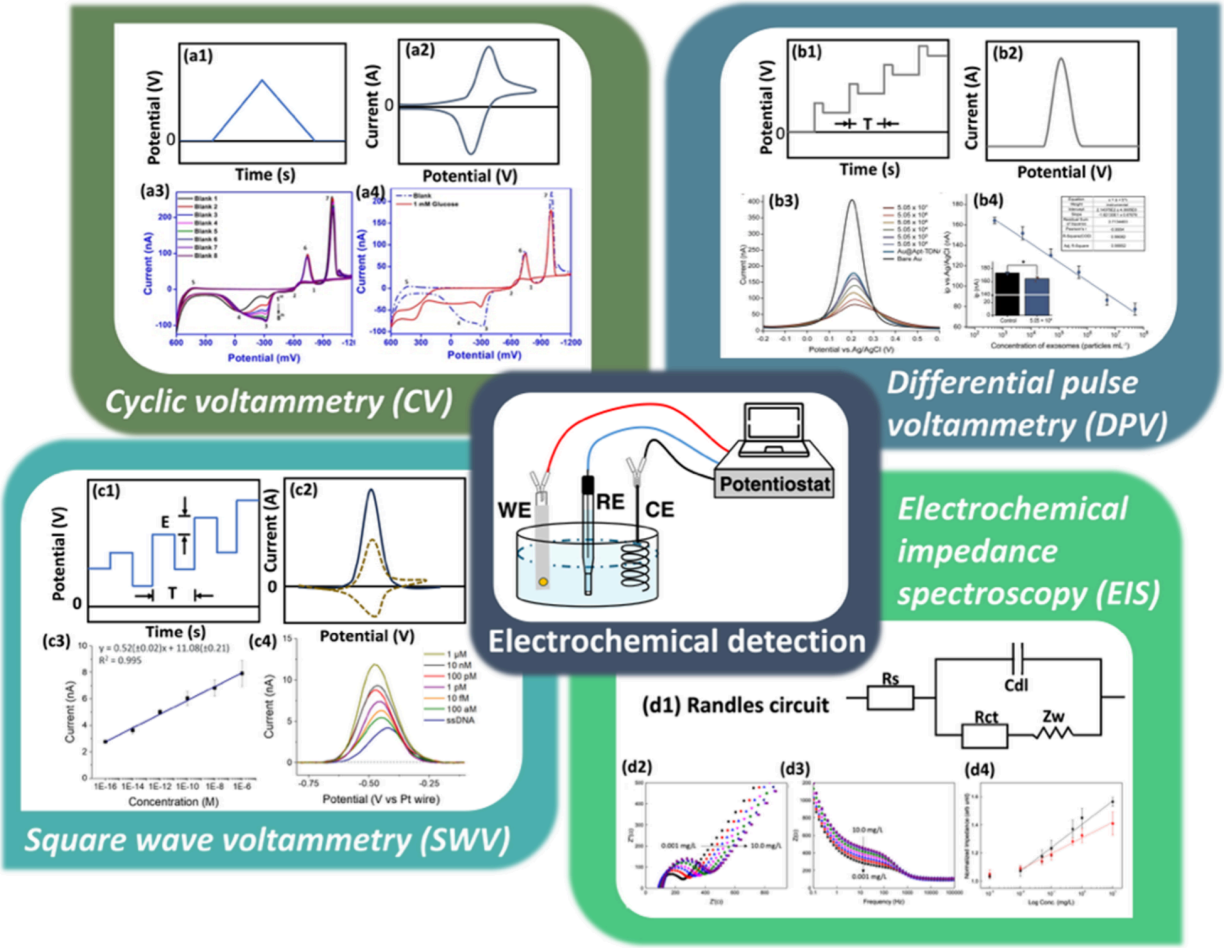
Common electrochemical techniques and examples: (a) cyclic voltammetry (CV), (b) differential pulse voltammetry (DPV), (c) square wave voltammetry (SWV), and (d) electrochemical impedance spectroscopy (EIS). (a1) Applied potential waveform of CV; (a2) voltammogram of CV; (a3) consecutive cyclic voltammograms recorded on the CuNPs/HD-CNT*f* microsensor in 0.1 M NaOH solution; (a4) cyclic voltammograms recorded on the CuNPs/HD-CNT*f* microsensor in the absence (dotted line) and presence (solid line) of 1 mM glucose in 0.1 M NaOH. Reproduced with permission from ref 38 Copyright 2021, Elsevier B.V. (b1) Applied potential waveform of DPV; (b2) voltammogram of DPV; (b3, b4) DPV curves at a series of exosome concentrations and the resulting calibration line based on a DNA-immobilized sensor. The limit of detection achieved approximately 6.27 × 10^1^ particles mL^–1^. Reproduced with permission from ref 39 Copyright 2021, the Authors. Published by Springer Nature. This is an open-access article under the terms of the Creative Commons Attribution License (CC BY 4.0). (c1) Applied potential waveform of SWV; (c2) voltammogram of SWV; (c3) calibration line representing the difference between peak height and current of ssDNA plotted against the logarithm of the target DNA concentration; (c4) square wave voltammograms corresponding to each concentration of the calibration curve. Reproduced with permission from ref 40 Copyright 2022, the Authors. Published by Elsevier. This is an open-access article under the terms of the Creative Commons Attribution License (CC BY 4.0). (d1) Randles circuit as an equivalent circuit model of EIS; (d2–d4) EIS detection of calmodulin on peptide-immobilized sensor, which is presented by (d2) Nyquist plot and (d3) Bode plot; (d4) normalized impedance before (black) and after (red) BSA blocking for calmodulin concentrations of 0.001, 0.01, 0.05, 0.1, 0.5, 1.0, and 10.0 mg/L. Reproduced with permission from ref 41 Copyright 2020, American Chemical Society.

### 3.1 Voltammetry

Voltammetry detects electroactive species by monitoring current responses as a function of applied potential, providing molecular selectivity based on characteristic redox potentials.^42^ In biosensing, changes in faradaic current can be directly correlated with analyte concentration or surface passivation caused by bioadhesion. Compared with purely mass-based techniques, voltammetric readouts offer inherently quantitative and target-specific information, making them well-suited for complex biological environments.

#### 3.1.1 Cyclic Voltammetry (CV)

In cyclic voltammetry (CV), a triangular potential waveform is applied to the working electrode (Figure 2a1), inducing alternating oxidation and reduction of electroactive species and generating a current response governed by coupled electron-transfer and mass-transport processes. The cyclic potential sweep enables continuous probing of both anodic and cathodic reactions, yielding characteristic oxidation and reduction peaks (Figure 2a2). The peak potentials provide direct information on redox thermodynamics, while the peak shapes and separations reflect reaction reversibility and kinetics.

Since the seminal theoretical treatment by Nicholson and Shain, which unified charge-transfer kinetics with homogeneous chemical reactions, CV has been widely adopted as a mechanistic tool in electrochemical biosensing to evaluate reaction pathways, rate-limiting steps, and electrode surface processes.^43^ Although CV generally offers lower sensitivity than pulse techniques such as DPV and SWV, its ability to resolve complex redox behavior makes it indispensable for mechanistic analysis and sensor characterization.

This capability is exemplified by the Cu nanoparticle–based glucose sensor developed by Gupta et al., where successive cyclic voltammograms recorded in 0.1 M NaOH revealed multiple redox processes associated with oxygen adsorption and the Cu(0)/Cu­(I)/Cu­(II) transitions (Figure 2a3).^38^ The appearance of seven distinct peaks, consistent with prior reports, enabled detailed assignment of surface and bulk electrochemical reactions.^44–46^ Moreover, comparison of voltammograms acquired in the absence and presence of glucose (Figure 2a4) demonstrated glucose oxidation–induced perturbations of copper and oxygen adsorption behavior. This example highlights that, despite the availability of highly sensitive pulse voltammetric techniques, CV remains irreplaceable for elucidating electrochemical reaction mechanisms and interfacial processes in biosensing systems.

#### 3.1.2 Linear Sweep Voltammetry and Pulse Voltammetry

Compared to the cyclic applied potential in CV, linear sweep voltammetry (LSV) applies a unidirectional potential scan to drive redox reactions at the working electrode. Differential pulse voltammetry (DPV) and square wave voltammetry (SWV) are pulse-modified derivatives of LSV that are specifically designed to enhance sensitivity by suppressing background noise.

In DPV, a series of potential pulses with fixed amplitude is superimposed onto a slowly increasing staircase potential (Figure 2b1). The current is sampled immediately before and at the end of each pulse, and the difference between these two values is calculated, effectively minimizing the contribution from non-Faradaic charging currents and amplifying the Faradaic response associated with analyte redox processes (Figure 2b2). SWV employs a staircase potential overlaid with symmetrical square pulses in both forward and reverse directions (Figure 2c1). The resulting anodic and cathodic currents are measured within each potential step, and the net current is obtained by subtracting the cathodic current from the anodic current (Figure 2c2).^42^

Due to their pulse-based operation, both DPV and SWV exhibit significantly improved signal-to-noise ratios compared to conventional LSV and CV, making them particularly suitable for detection that requires low detection limits. SWV generally enables faster measurements due to its shorter potential periods and often achieves higher sensitivity than DPV.^42,47^ However, the high-frequency potential modulation in SWV may overlook asymmetric diffusion-controlled responses. It may be less suitable for sluggish or irreversible electrochemical reactions.^48^ Accordingly, SWV is commonly favored for rapid detection and reversible systems. In contrast, DPV is better suited for slower kinetics and irreversible biosensing reactions.

The analytical advantages of DPV are exemplified by Jiang et al., who employed DPV in a polydopamine-assisted, aptamer-functionalized tetrahedral DNA microelectrode for exosome detection, achieving single-particle sensitivity with a limit of detection of approximately 6.27 × 10^1^ particles mL^–1^ (Figure 2b3,b4).^39^ In contrast, the high temporal resolution of SWV has been leveraged by Wasiewska et al. for amplification-free DNA sensing of *stx1* genes from Shiga toxin–producing *E. coli* , achieving a linear response between 10^–16^ and 10^–6^ M of synthetic target strand with the lowest measured limit of detection of 100 aM after 20 min (Figure 2c3,c4).^40^

### 3.2 Electrochemical Impedance Spectroscopy (EIS)

Unlike amperometry and voltammetry, which directly measure current or potential responses, electrochemical impedance spectroscopy (EIS) probes the frequency-dependent response of an electrochemical system to a small sinusoidal potential perturbation. By analyzing the resulting complex impedance, *Z*(ω), which comprises both resistive and capacitive components, EIS provides rich information on interfacial processes, including charge transfer, double-layer capacitance, and mass transport. This frequency-domain approach enables the investigation of intrinsic material properties, surface modification states, and biorecognition events occurring at electrode interfaces.^8^

Interpretation of EIS data typically relies on fitting the experimental spectra with appropriate equivalent circuit models. Among them, the Randles circuit is the most widely adopted framework, consisting of solution resistance (*R*_s_), charge-transfer resistance (*R*_ct_), double-layer capacitance (*C*_dl_), and diffusion-related impedance (*Z*_w_) (Figure 2d1). EIS responses are commonly visualized using Nyquist plots (Figure 2d2), which depict the imaginary component versus the real component of impedance, and Bode plots (Figure 2d3), which present the impedance magnitude and phase as functions of frequency. In biosensing applications, analyte binding or surface fouling often manifests as an increase in *R*_ct_ accompanied by a decrease in *C*_dl_, leading to an enlarged semicircle in the Nyquist plot.^49,50^

**Figure 3:**
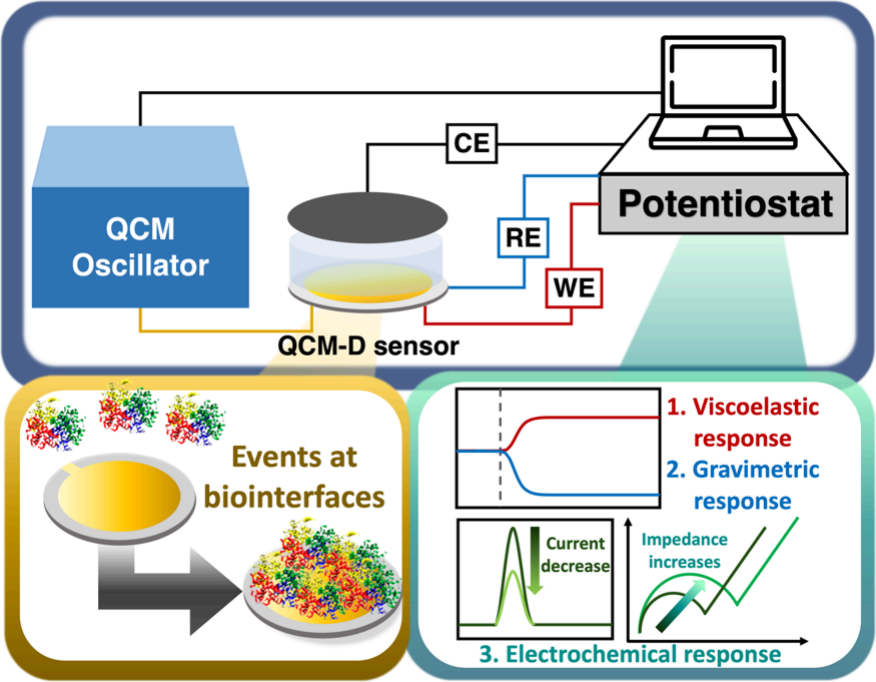
Scheme of the EQCM-D instrument and the ability to simultaneously record viscoelastic, gravimetric, and electrochemical responses.

Owing to its sensitivity to interfacial changes, EIS has become one of the most widely employed electrochemical techniques in biosensing. Notably, EIS can be implemented in a label-free configuration, eliminating the need for redox probes or enzyme labels and thereby simplifying sensor fabrication while minimizing potential interference from ions or catalytic species. For instance, Guru and co-workers employed label-free EIS to detect lysed exosome-derived proteins through changes in surface capacitance.^51^ Chin et al. reported an electrochemical aptasensor for calmodulin detection using EIS. The Nyquist plots and Bode plots (shown in Figure 2d2–d4) indicate the impedance change with different concentrations of calmodulin.^41^

In practice, electrochemical techniques are often combined to obtain complementary information. Wasiewska et al. developed an amplification-free multiplex electrochemical sensor for the simultaneous detection of *stx1* and *stx2* genes, where CV and EIS were employed to monitor electrode modification and interfacial properties. At the same time, SWV enabled rapid and highly sensitive quantification of the target. This integrated strategy achieved an ultralow limit of detection of 100 zM for both genes.^52^

### 3.3 Limitations of Electrochemical Techniques

Despite their high sensitivity and versatility, electrochemical techniques are inherently influenced by experimental conditions, including scan rate, temperature, and solution pH, as implied by fundamental electrochemical relationships such as the Nernst and Randles–Ševčík equations.^53,54^ Variations in these parameters can alter reaction kinetics, mass transport, and interfacial charge distribution, thereby affecting signal magnitude, selectivity, and reproducibility. For instance, Wu et al. demonstrated that pH-dependent electrochemical responses significantly impact the discrimination of dopamine from common interferents such as ascorbic acid and uric acid.^55^ Similarly, Zinatloo-Ajabshir et al. reported that scan rate and pH strongly modulate the differential pulse voltammetric response and sensitivity of a mesalazine sensor.^56^

Beyond these general factors, electrochemical impedance spectroscopy (EIS) faces additional limitations arising from its reliance on equivalent circuit modeling. Quantitative interpretation of EIS spectra requires the selection of appropriate circuit elements and fitting strategies to represent complex interfacial processes. However, in practice, equivalent circuits are sometimes empirically constructed to reproduce experimental spectra without a rigorous physical basis, potentially decoupling the extracted parameters from the actual electrochemical behavior of the electrode interface.^57^ This model dependence complicates data interpretation and limits the comparability of EIS results across different sensing platforms.

## 4 Coupling of QCM-D and Electrochemical Techniques

Since QCM-D and electrochemical techniques transduce interfacial processes through fundamentally different physical mechanisms, their combination has been increasingly adopted to enable a more comprehensive interrogation of complex biointerfaces. QCM-D is inherently sensitive to mass loading, hydration, and viscoelastic properties of surface-bound layers, whereas electrochemical techniques primarily report on charge transfer, interfacial accessibility, and local electrochemical environments. Probing the same interfacial events from these orthogonal perspectives allows not only cross-validation of sensing signals but also deeper mechanistic interpretation of adsorption, binding, and structure at electrode surfaces.

Practically, the specific roles assigned to QCM-D and electrochemical methods vary across studies. Table 1 provides a clear methodological overview, the key features, advantages, and limitations of major types of coupling strategies for QCM-D and EC techniques, including the direct integration (EQCM-D) which will be addressed in the next section. In many cases, QCM-D is employed to monitor the construction of the sensing platform, while electrochemical techniques provide rapid and quantitative detection. For example, In Chin et al.’s work mentioned above, QCM-D was used to resolve the dynamic binding processes between sensing probes and CaM, thereby validating the formation of a functional interface before electrochemical impedance spectroscopy (EIS) detection.^41^ Similarly, Ding et al. combined CV, QCM-D, and EIS to characterize sensor fabrication and achieve quantitative detection of *Toxoplasma gondii* -specific IgG, highlighting how complementary techniques can collectively strengthen analytical reliability.^58^

**Table 1:** Methodological Integration and Strategic Advantages of Representative QCM-D and Electrochemical Platforms

coupling mode	representative work	analyte	QCM/QCM-D	EC technique	advantage	key limitation
parallel/sequential	Chin et al.^41^	protein (calmodulin)	monitoring the binding process	EIS: quantitative detection	LoD: 0.001 mg/L.	1. risk of sample inconsistency between channels.
	Ding et al.^58^	protein (IgG)	validation for the protein binding	EIS: quantitative detection	LoD: 1:9600 dilution.	
	Gao et al.^59^	protein (CRP)	quantitative detection	CV: validation for the platform buildup	LOD: 0.02 μg/mL	2. nonsynchronous; no real-time correlation.
EQCM-D	Yang et al.^60^	cell (erythrocyte)	quantitative detection	CV: validation for the platform buildup	in situ analysis of sialic acid (SA) on the cell surface	1. electrochemical perturbations due to solvent and electrolyte interaction that influence gravimetric responses
	Suthar et al.^61^	exosome	studying the binding dynamics and structural change	EIS: quantitative detection	LoD: 6.71 × 10^7^ particles/mL in 25% v/v serum	2. complex interpretation of soft/heterogeneous layers (e.g., cells/vesicles) due to viscoelastic model assumptions.
	Lin et al.^62^	water	water dynamics under the influence of surface properties, added salts, and applied potentials		robust evidence for water behavior	
EQCM	Kanyong et al.^63^	electrodeposition layer	quantitative study for electrodeposition		evaluation of grafting efficiency, molecular surface coverage, and film thickness	

Conversely, electrochemical techniques may serve as the primary tool for confirming surface modification or target capture. At the same time, QCM-D provides label-free detection or additional insight into mass and mechanical changes. Gao et al. demonstrated a sensitive C-reactive protein sensor based on QCM-D detection. In their work, QCM-D was responsible for quantitative detection while CV monitoring the buildup of the sensing platform.^59^ These examples illustrate that QCM-D and electrochemical detection are not constrained to fixed analytical roles but can be flexibly deployed depending on the sensing objective.

The agreement between mass-based and charge-based signals enhances confidence in detection. At the same time, discrepancies between the two can reveal solvent coupling, viscoelastic effects, or changes in interfacial electron-transfer pathways that would remain ambiguous if only one technique were adopted. Such multimodal cross-validation is particularly crucial for soft and hydrated biological systems, where single-parameter readouts often fail to capture the full complexity of interfacial phenomena.

## 5 Integration of QCM-D and Electrochemical Detection—EQCM-D

In addition to the sequential or parallel coupling of QCM-D and electrochemical techniques, a more advanced strategy is their integration into a single platform, termed electrochemical quartz crystal microbalance with dissipation monitoring (EQCM-D). In EQCM-D, the QCM sensor simultaneously serves as the working electrode in a conventional three-electrode electrochemical cell, enabling synchronous gravimetric, viscoelastic, and electrochemical measurements at the same interface and under identical experimental conditions (Figure 3).^61^ This simultaneous and colocalized transduction fundamentally distinguishes EQCM-D from conventional cross-validation approaches, as it minimizes uncertainties arising from sensor-to-sensor variations and temporal mismatches, and allows direct correlation between frequency, dissipation, and electrochemical signals in real time.^59^

The integration of QCM and electrochemistry dates back to 1985, when Bruckenstein and Shay described a practical circuit for using a QCM with one electrode in contact with an electrolyte solution and subject to an electrochemical control function.^16^ This is considered the beginning of the EQCM technique. Since then, EQCM has been applied to electrochemical reactions and electrode modifications, including electrocatalysis, (photo)­electrocatalysis, supercapacitors, batteries, corrosion electrochemistry, and electrodeposition.^64–66^ The introduction of dissipation monitoring in the late 1990s marked a critical transition of the integration of QCM and EC techniques (so-called “EQCM-D”) toward biological applications, as it enabled the differentiation between rigid mass loading and soft, hydrated, and viscoelastic contributions that dominate biointerfaces.

An immediate advantage of EQCM-D lies in its ability to simultaneously record gravimetric, viscoelastic, and electrochemical responses at the same interface, thereby improving analytical robustness by reducing uncertainties associated with sensor-to-sensor variability and temporal mismatch. From a technical perspective, the integration of a potentiostat and its associated electrical connections may unavoidably introduce instrumental noise, such as parasitic capacitance^67^ or thermal perturbation,^68^ which could marginally elevate the baseline fluctuations of Δ*F* and Δ*D*. However, these hardware-related trade-offs are often outweighed by the significant analytical gains of synchronized data acquisition. Suthar et al. reported a direct immunosensing approach using EQCM-D for label-free detection of plasma exosomes. By integrating QCM-D with in situ EIS measurements, they demonstrated correlations between mass loading, viscoelasticity, and impedance responses across different functional layers and analytes, resulting in a 2–4-fold improvement in the limit of detection (6.71 × 10^7^ exosome-sized particles per mL in 25% v/v serum).^61^ This enhancement highlights that by correlating multiple signal modalities in real-time, EQCM-D can achieve a superior analytical LOD compared to independent measurements, effectively compensating for the increased instrumental complexity. Similarly, Yang et al. demonstrated an erythrocyte sensor in which CV revealed peak shifts and current suppression associated with erythrocyte capture, while QCM-D directly quantified the corresponding real-time adsorption processes.^60^

In addition to improving analytical robustness, EQCM and EQCM-D enable direct comparison between mass uptake and electrochemical charge transfer, offering mechanistic insight into interfacial processes that cannot be accessed by either technique alone. Kanyong et al. employed an EQCM coupled with in situ infrared spectroscopic ellipsometry to quantitatively study the *p*-maleimidophenyl (pMP) electrodeposition.^63^ The integration of CV and QCM enables the simultaneous observation of the current response and frequency shift caused by each cycle of pMP electrodeposition. The team further calculated the mass changes, Δ*m*_QCM_ and Δ*m*_charge_, from both parts via Faraday’s law and the Sauerbrey equation. With the known information, such as the electrode area and the molecular weight of pMP, the team assessed the evaluation of grafting efficiency, molecular surface coverage, and film thickness.

Compared to individual QCM-D and electrochemical methods, EQCM-D is particularly well-suited for deep insight into water behaviors, which play an active role in soft and hydrated biological systems rather than just serving as a passive background.^69,70^ Lin et al. conducted EQCM-D to evaluate the interactions between water and the electrode surface.^62^ The real-time changes in frequency and dissipation indicated by EQCM-D results provide robust evidence for water behaviors under the influence of surface properties, added salts, and applied potentials.

## 6 Perspective

EQCM-D has demonstrated clear advantages over the sequential or parallel coupling of individual QCM-D and electrochemical techniques in biosensing applications. Nevertheless, EQCM-D not only integrates the strengths of gravimetric and electrochemical transduction but also inevitably inherits their intrinsic limitations. As discussed above, electrochemical measurements require careful control over environmental parameters such as temperature and pH. In addition, similar to conventional QCM measurements, the requirement for relatively large sample volume remains a practical concern, which is expected to be addressed in the near future with the development of microfluidic techniques.

Beyond these inherited constraints, a more fundamental challenge arises from the fact that gravimetric and electrochemical responses in EQCM-D are no longer independent of each other. While QCM-D and electrochemical techniques are often regarded as complementary and orthogonal, their direct integration tends to cause mutual interference that cannot be neglected. In the following sections, we focus on interpretation challenges originating from this coupling, with particular emphasis on the active roles of water molecules and ions at electrochemical interfaces. Future perspectives on correlative integration with spectroscopic and microscopic techniques are also briefly discussed.

### 6.1 Active Roles of Water and Ions: Challenges in Data Interpretation

EQCM-D data are frequently interpreted under the assumption that gravimetric and electrochemical signals provide independent and complementary information. In practice, however, electrochemical perturbations can directly influence QCM-D responses. The external potential or current induces ion transport and the formation of an electric double-layer (EDL) at the electrode/electrolyte interface, which can substantially modify the local viscoelastic environment sensed by the oscillating quartz crystal. For example, Tsionsky et al. demonstrated that EDL formation can significantly alter the viscoelastic properties near the electrode surface, hence overshadowing the mass and dissipation contributions arising from ion adsorption alone.^71^

The growing interest in interfacial water behavior further highlights the complexity of EQCM-D signal interpretation. Several studies have shown that hydrated ions and structured interfacial water layers can contribute to interfacial capacitance.^72,73^ Notably, such water-associated effects become significantly more pronounced when the electrode surface is functionalized with hydrophilic self-assembled monolayers (SAMs) or polymer brushes. The former can establish a well-defined hydrated interface, while the latter, due to the softness and hydrophilicity, can entrap a large volume of solvent, leading to distinct dissipation signatures that reflect the dynamic coupling between surface modification and interfacial hydration.^74,75^ These water-associated effects may induce significant changes in frequency, dissipation, and capacitance, in some cases exceeding the responses directly attributed to biomolecular adsorption. Moreover, recent work by Conner and Holubowitch revealed that during the electrochemical deposition of conjugated polymer films, electrolyte species can become trapped within the films as dopants.^76^ Although these dopants are essential for electrochemical redox signals and label-free detection, their insertion and expulsion cause strong background contributions to EQCM signals. This disturbance further complicates quantitative interpretation and may ultimately reduce analytical reliability.

Because contributions from water molecules and ions are intrinsic to electrochemical interfaces, they cannot be readily eliminated. Furthermore, common viscoelastic models (e.g., Kelvin–Voigt) often assume a laterally homogeneous and continuum layer, which is rarely satisfied for discrete and structurally complex biological assemblies like cells or extracellular vesicles. These assumptions may lead to simplified interpretations that overlook localized hydration dynamics and mechanical remodeling. Transitioning toward physically consistent models remains an urgent challenge.

Instead, rigorous and physically consistent interpretation becomes essential. To date, however, no unified theoretical framework has been established that can simultaneously and quantitatively describe QCM-derived gravimetric and viscoelastic parameters, as well as electrochemical observables such as impedance responses or charge-transfer processes. The absence of comprehensive models remains an urgent challenge to the quantitative application of EQCM-D in complex biological systems.

### 6.2 Strategies for Signal Validation and Deconvolution

To transition EQCM-D from a qualitative observation tool to a rigorous analytical platform, systematic validation frameworks are needed to deconvolve the intertwined signals. Herein, strategies based on kinetic scales and overtone dissipation are raised for validating genuine biological recognition against electrochemical artifacts (e.g., EDL restructuring and ion-induced hydration changes).

#### 6.2.1 Temporal Discrimination Based on Kinetic Scales

A primary strategy for signal deconvolution is the analysis of characteristic response time scales. During EQCM-D measurements, electric double-layer (EDL) restructuring and ion transport typically reach equilibrium within seconds, resulting in near-instantaneous fluctuations in frequency and dissipation.^77–79^ In contrast, biorecognition events, such as protein tethering or exosome capture, typically evolve over several minutes to hours before stabilizing in the QCM-D profile.^27^ This temporal mismatch allows researchers to decouple cumulative biological mass uptake from transient electrochemical fluctuations, a distinction that is particularly critical when identifying low-abundance targets like exosomes in complex matrices.

#### 6.2.2 Overtone Dispersion as a Viscoelastic Fingerprint

The multiharmonic nature of QCM-D offers a unique mechanism for spatial deconvolution based on the overtone-dependent penetration depth (δ). According to the relationship following equation:

$$\delta ={\left(\frac{\eta_{m}}{\pi nf_{0}\rho_{m}}\right)}^{1/2}$$

where ρ_m_ and η_m_ are the density and the viscosity of the deposited material, and *f*_0_ is the fundamental frequency of the QCM-D sensor (4.95 MHz for the Au-coated quartz crystal),^80^ the sensing volume contracts toward the electrode surface as the overtone (*n*) increases. This physical property can be strategically utilized to differentiate surface-confined electrochemical artifacts from extended biological binding events. For a typical 4.95 MHz crystal in aqueous media, the penetration depth ranges from approximately 145 nm for *n* = 3–110 nm for *n* = 5, and drops further for higher harmonics. Considering that electric double-layer (EDL) restructuring and ionic flux are primarily confined to the immediate vicinity of the electrode (typically <10 nm), these electrochemical effects will manifest across all measurable overtones. However, large biological targets, such as exosomes or cellular interfaces, extend significantly further into the solution. By comparing the responses of lower-order overtones (which probe the entire biological layer) with higher-order overtones (which are more sensitive to the basal attachment and ionic background), researchers can isolate specific binding signatures. As exemplified by Rogala et al., selecting the seventh overtone allowed for precise monitoring of a 27 nm cell-adhesion layer while minimizing interference from bulk solvent fluctuations. Such depth-matching strategies ensure that the recorded frequency and dissipation shifts reflect the structural evolution of the target biointerface rather than localized ionic rearrangements.^81^

## 7 Future Outlook: From Multimodal EQCM-D Platforms to Cellular Responses to Electrical Stimulation

To disentangle the intertwined gravimetric, viscoelastic, and electrochemical responses in EQCM-D, an increasing number of studies have adopted correlative multimodal platforms by combining spectroscopic and microscopic techniques, including infrared spectroscopy,^62,63^ Raman spectroscopy,^82^ atomic force microscopy,^83,84^ and X-ray photoelectron spectroscopy.^85^ These complementary methods provide independent morphological, structural, or chemical fingerprinting information that assists in the interpretation of EQCM-D signals. These multimodal strategies have been most extensively applied in nonbiological electrochemical systems, particularly in battery and energy-storage research. Their extension to biological and bioelectrochemical interfaces is therefore highly expected for simultaneous interrogation of biomolecular adsorption, hydration structure, and interfacial charge-transfer processes.

Importantly, the integration of additional techniques should not be viewed as merely accumulating data. Instead, correlative multimodal platforms offer a pathway to mechanism-driven interpretation, allowing EQCM-based systems to move beyond phenomenological descriptions toward quantitative and physically consistent models of biointerfaces.

Looking forward, an emerging and promising direction for EQCM-D is to further investigate cellular responses to external electrical stimulation. External potentials and currents are known to regulate ion-channel activity, membrane polarization, cellular adhesion, and metabolic behavior across a wide range of biological systems, including neurons, muscle cells, and electroactive bacteria. Integrating controlled electrical stimulation with EQCM-D may enable real-time correlation between electrical inputs and cellular feedback, which is reflected by gravimetric and viscoelastic response, hydration dynamics, and interfacial electrochemical responses. Such platforms may enable a deeper understanding of cellular sensing and adaptation to electrical environments, opening new avenues for the development of bioelectronic interfaces, microbial fuel cells, electrically assisted tissue engineering, and emerging electroceutical applications.
